# Lupus Disguised as Chorea: Uncommon Presentation of a Common Disease

**DOI:** 10.1002/ccr3.70376

**Published:** 2025-03-27

**Authors:** G. C. Bhumika, Shrijana Shrestha, Firoz Anjum, Shreya Thapa, Rahul Shrestha

**Affiliations:** ^1^ Department of Pediatrics Patan Academy of Health Sciences Patan Nepal; ^2^ Department of General Practice and Emergency Medicine Patan Academy of Health Sciences Patan Nepal

**Keywords:** chorea, lupus, neuropsychiatric lupus, pediatrics

## Abstract

This case report presents a preadolescent female with a rare presentation of lupus as isolated chorea. An investigation to find out the cause of unexplained cytopenia with raised inflammatory markers ultimately led to the diagnosis of neuropsychiatric lupus with antiphospholipid antibodies. Lupus should always be included in the differential for chorea in children.

## 1 Introduction

Lupus is a chronic autoimmune inflammatory disease of unknown cause that can affect any organ system. Although Lupus in children is fundamentally the same disease as in adults, with similar etiology, pathogenesis, and laboratory findings, there are some differences in the frequency and severity of certain clinical manifestations [1].

In 1999, the American College of Rheumatology issued a proposal for the nomenclature and definition of Neuropsychiatric Lupus (NPSLE). The proposal defined 19 syndromes, including movement disorders [1]. Although NPSLE is documented in a range of 13.5%–34.6% of pediatric patients diagnosed with Lupus [2, 3], chorea is a rare neurologic syndrome associated with it, described in 1%–3% of patients with Lupus. While the predominant cause of chorea in children is typically the autoimmune variant resulting from post‐streptococcal origins, it is important to note that chorea can also arise as a complication of Lupus [4].

We report a case of acute onset chorea as a form of Lupus.

## 2 Case History

A preadolescent female presented to the emergency department of a tertiary care center in Nepal with random dance‐like involuntary movements affecting the limbs, which gradually increased in frequency and severity over the course of 2 weeks. The movements initially involved her left upper and lower limbs, later affected all four limbs, but were not associated with any changes in the level of consciousness and were notably absent when asleep. Her history was insignificant except for a sore throat and fever for a few days, 4 months prior, which resolved without treatment. She did not have any history of altered sensorium, seizures, degrading school performance, loss of consciousness, incontinence, fever, vomiting, headache, joint pain, weight loss, rashes, ulcers, or toxin exposure.

On admission, she was a well‐oriented, well‐built prepubertal girl with generalized choreiform movements, a darting tongue, milkmaid's grip, pronator drift, and spooning sign. The remaining general and systemic examinations were normal.

## 3 Methods

Initial workup revealed bicytopenia (low hemoglobin for her age and leukocytopenia) with a borderline platelet count and an increased erythrocyte sedimentation rate as shown in Table 1.

**TABLE 1:** Results of initial laboratory investigations.

Parameters	Normal values	Values
Total leukocyte count (/cubic millimeter)	4000–11,000	3590
Differential count	Neutrophils 40%–75% Lymphocytes 20%–40%	70% Neutrophils 30% Lymphocytes (Absolute lymphocyte count 1077)
Hemoglobin (g/dL)/hematocrit(%)	12–15/34–46	11/33
Platelet count (/cubic millimeter)	150,000–400,000	166,000
Erythrocyte sedimentation rate (mm/h)	Upto 20	87
Antistreptolysin O	Negative	Negative
Urea/creatinine (mg/dL)	15–45/0.3–0.8	44/0.6
Sodium/potassium (mmol/L)	135–145/3.5–5	138/4.3

After the initial evaluation, she was started on Haloperidol 0.25 mg three times daily to manage the disabling movement disorder.

The bicytopenia revealed in the laboratory test results led to further workup in the line of an autoimmune disorder which showed an elevated anti‐nuclear antibody (ANA) titer of 1:640 (Normal range (NR) < 1: 40), a positive anti‐double‐stranded DNA, positive anti‐histone antibodies with hypocomplementemia—low C3 and C4, C3 39 mg/dL (NR 83–193) and C4 11.5 mg/dL (NR 15–57). Urinalysis, thyroid function test, electrocardiogram, and chest X‐ray were normal. Echocardiography showed normal mitral leaflets with trace aortic and mitral regurgitation. MRI brain was performed which showed a few T2 flair, high signal punctate foci in bilateral frontal lobes, that is, nonspecific demyelinating foci. Further investigation showed positive anticardiolipin IgG and antibeta 2 glycoprotein IgG antibody in the titres of 418 U/mL and 312 U/mL respectively.

After having all the available investigation results, we scored her on the EULAR 2019 classification criteria for lupus [1]. With features of neurological involvement (chorea), leukopenia, low C3, positive anti‐dsDNA and antiphospholipid antibodies, the patient scored 14 in the EULAR 2019 classification criteria [1]. The patient was then diagnosed as NPSLE and was started on hydroxychloroquine, azathioprine, and prednisolone. Aspirin was added to her treatment and was planned for repeat antiphospholipid antibody titers after 12 weeks. As the choreiform movements gradually decreased and ceased after the first few days of hospitalization, haloperidol was tapered off.

## 4 Conclusions/Outcome

She is doing well on medications, is currently asymptomatic, and has resumed school.

## 5 Discussion

This case report presents a preadolescent girl whose lupus diagnosis was revealed by a new onset chorea as a part of the acute neurological manifestation of lupus, along with bicytopenia, elevated titers of anti‐dsDNA antibodies, positive antinuclear antibodies, and positive antiphospholipid antibodies.

While chorea can manifest as a symptom in various diseases, when a child exhibits generalized chorea as the sole symptom, Sydenham's chorea is typically the initial diagnosis in regions including Nepal with a high prevalence of Rheumatic Heart Disease [5]. The observation of leukopenia, lymphopenia, and an elevated erythrocyte sedimentation rate (ESR) raised concerns, leading to the search for an alternative diagnosis that could encompass the multisystem manifestations. Hence, the investigations for autoimmune disease were performed, ultimately revealing a diagnosis of lupus.

Lupus is a common diagnosis in the pediatric population. It mostly presents in the form of lupus nephritis [6]. The American College of Rheumatology (ACR) nomenclature for neuropsychiatry syndromes in lupus includes 12 central nervous system syndromes and 7 peripheral nervous system syndromes. There is a wide variation in the frequency of NPSLE in various studies. Mild cognitive dysfunction and mood disorders are frequently seen as manifestations of NPSLE (6%–80%) whereas movement disorders including chorea are extremely rare (< 1% of lupus patients) [7].

Neuropsychiatric (NP) manifestations are a rare presentation of Lupus. In a study carried out in Turkey among 1107 juvenile lupus patients, 149 patients had NP involvement (13.5%). The most common NPSLE findings were headache (50.3%), seizure (38.3%), and acute confusional state (33.6%). A study found the prevalence of movement disorders in lupus to be 9.4% [8].

Chorea as the sole initial presentation of lupus remains rare across all age groups. It manifests in only around 1%–3% of cases [9].

The exact mechanism behind lupus‐associated chorea remains uncertain, but it is potentially linked to damage in the vascular, neuronal, and glial components. There is a high incidence of antiphospholipid antibodies (aPL) in studies of lupus patients with neuropsychiatric manifestations, between 25% and 90%, perhaps implicating a role of these antibodies in the pathogenesis [10]. Vascular injury may involve both inflammatory and thrombotic processes. The presence of aPL appears to have a significant impact on the pathogenesis of NPSLE. The presence of LAC is strongly associated with stroke, transient ischemic attack, transverse myelitis, epilepsy, chorea, and dementia. Although there are no disease‐specific antibodies for NPSLE, the presence of Anti SSA, Anti SSB, and Anti ribosomal P antibodies (Anti‐RibP) correlates with the disease [11].

The European League Against Rheumatism (EULAR) recommends antiplatelet therapy for lupus patients with movement disorders and positive aPL antibodies. Anticoagulation agents are only suggested for patients with thrombotic manifestations. As there are no specific guidelines for children with NPSLE, the management should be individualized and based on the decision of the treating clinician [12].

As chorea is a frequent manifestation of rheumatic fever, the diagnosis of NPSLE can be delayed, particularly in regions where rheumatic fever is widespread. Despite its rarity, it is essential to consider the possibility of childhood lupus in the differential diagnosis of any child presenting with unexplained neurological symptoms.

## Author Contributions

**G. C. Bhumika:** conceptualization, investigation, writing – original draft, writing – review and editing. **Shrijana Shrestha:** supervision, writing – review and editing. **Firoz Anjum:** investigation. **Shreya Thapa:** data curation. **Rahul Shrestha:** methodology.

## Consent

Written informed consent was taken from the patient's father in accordance with the journal's policy.

## Conflicts of Interest

The authors declare no conflicts of interest.

## Data Availability

The data that support the findings of this study are openly available in Clinical case reports, reference number CCR3‐2024‐09‐2936.

## References

[1] M. Aringer , K. Costenbader , D. Daikh , et al., “2019 European League Against Rheumatism/American College of Rheumatology Classification Criteria for Systemic Lupus Erythematosus,” Arthritis and Rheumatology 71, no. 9 (2019): 1400–1412. 31385462 10.1002/art.40930 PMC6827566

[2] A. P. Kısaarslan , S. Ö. Çiçek , E. D. Batu , et al., “Neuropsychiatric Involvement in Juvenile‐Onset Systemic Lupus Erythematosus: A Multicenter Study,” Joint, Bone, Spine 90, no. 4 (2023): 105559. 36858168 10.1016/j.jbspin.2023.105559

[3] H. H. Yu , J. H. Lee , L. C. Wang , Y. H. Yang , and B. L. Chiang , “Neuropsychiatric Manifestations in Pediatric Systemic Lupus Erythematosus: A 20‐Year Study,” Lupus 15, no. 10 (2006): 651–657. 17120591 10.1177/0961203306070990

[4] F. Cardoso , “Difficult Diagnoses in Hyperkinetic Disorders—A Focused Review,” Frontiers in Neurology 3 (2012): 151, 10.3389/fneur.2012.00151. 23112789 PMC3482700

[5] P. R. Regmi , A. Adhikaree , U. Bhattarai , et al., “Rheumatic Heart Disease in School‐Attending Nepalese Children: A Descriptive Analysis of the National Heart Screening Database,” Indian Heart Journal 75, no. 5 (2023): 363–369. 37495016 10.1016/j.ihj.2023.07.003 PMC10568058

[6] R. Mina and H. I. Brunner , “Pediatric Lupus—Are There Differences in Presentation, Genetics, Response to Therapy, and Damage Accrual Compared With Adult Lupus?,” Rheumatic Disease Clinics of North America 36, no. 1 (2010): 53–80. 20202591 10.1016/j.rdc.2009.12.012 PMC2837537

[7] M. Govoni and J. G. Hanly , “The Management of Neuropsychiatric Lupus in the 21st Century: Still So Many Unmet Needs?,” Rheumatology 59, no. Supplement_5 (2020): v52–v62. 33280014 10.1093/rheumatology/keaa404 PMC7719041

[8] F. P. S. T. Santos , B. R. Nascimento , D. C. Calderaro , G. A. Ferreira , and H. Correa , “Neuropsychiatric Syndromes in Childhood‐Onset Systemic Lupus Erythematosus: A Systematic Review and Meta‐Analysis,” Journal of Clinical Rheumatology 27, no. 5 (2021): 206–214. 31022053 10.1097/RHU.0000000000001029

[9] Y. Arnson , Y. Shoenfeld , E. Alon , and H. Amital , “The Antiphospholipid Syndrome as a Neurological Disease,” Seminars in Arthritis and Rheumatism 40, no. 2 (2010): 97–108. 19596138 10.1016/j.semarthrit.2009.05.001

[10] K. Kyle , Y. Bordelon , N. Venna , and J. Linnoila , “Autoimmune and Paraneoplastic Chorea: A Review of the Literature,” Frontiers in Neurology 13 (2022): 829076. 35370928 10.3389/fneur.2022.829076 PMC8972589

[11] J. F. Baizabal‐Carvallo and F. Cardoso , “Chorea in Children: Etiology, Diagnostic Approach and Management,” Journal of Neural Transmission 127, no. 10 (2020): 1323–1342. 32776155 10.1007/s00702-020-02238-3

[12] A. Fanouriakis , M. Kostopoulou , J. Andersen , et al., “EULAR Recommendations for the Management of Systemic Lupus Erythematosus: 2023 Update,” Annals of the Rheumatic Diseases 83, no. 1 (2024): 15–29. 37827694 10.1136/ard-2023-224762

